# The Effect of Corporate Social Responsibility Characteristics on Employee Green Behavior: A Moral Emotions Perspective

**DOI:** 10.3390/bs14111045

**Published:** 2024-11-05

**Authors:** Na Zhang, Xiaoyu Ren, Xiang Sun, Chunhua Jin

**Affiliations:** School of Economics and Management, Beijing Information Science and Technology University, Beijing 102206, China; b15033@buaa.edu.cn (N.Z.);

**Keywords:** employee green behavior, corporate social responsibility characteristics, moral emotion, organizational pride, environmental passion climate

## Abstract

Shaping green employees is a source of sustainable competitive advantage for enterprises. However, previous studies have lacked consideration of the moral aspects of employee green behavior (EGB), thus ignoring the mechanism of employee moral psychology, especially the important impact of moral emotions on EGB. Based on the affective events theory, we built a moderated mediation model to test how corporate social responsibility characteristics (CSRCs) affect EGB and to explore the role of organizational pride (OP) and the environmental passion climate (EPC) in this process. SPSS25.0 and Mplus8.0 were used to analyze data from 267 valid questionnaires. The results show that CSRCs’ positive effect required EGB and OP to play a mediating role in the relationship between them. In addition, EPC had a negative moderating effect on the effect of OP on required EGB. These results expand the theoretical territory of EGB from the perspective of moral emotions. In practice, the results provide a moral motivation for cultivating employees’ ecological literacy and stimulating EGB as well as management strategies for organizational environmental protection practice and green sustainable development.

## 1. Introduction

Organizational environmental management serves the “dual carbon” development strategic goals, which can improve organizational environmental performance and add a green background and quality to build a prosperous society in a comprehensive manner. As the subjective actors in the process of environmental management, employees are the undertakers and promoters of organizational environmental responsibility [1]. Employee green behavior (EGB) is related to the implementation effect of various environmental protection measures. In addition, EGB complements the green development strategy at the organizational level and green human resource management at the functional level. This forms a virtuous circle of sustainable development for corporate green development [2]. Therefore, EGB is defined as scalable action and behavior that employees engage in that are linked with and contribute to or detract from environmental sustainability [3]. Research on the motivation of EGB has become an important topic in achieving organizational environmental management goals and promoting organizational sustainable development [4,5].

Employees’ behavior is closely related to the organizational environment. Organizational rules and regulations, culture, climate, and organizational behavior are important factors affecting employee attitudes and shaping employees’ behavior [6]. Corporate social responsibility characteristics (CSRCs) are an aspect of organizational characteristics, which describe the long-term, stable organizational characteristics of corporate social responsibility from three dimensions: corporate social responsibility culture, corporate social responsibility climate, and corporate social responsibility performance (i.e., “thoughts”, “words”, and “actions”) [7]. Compared with the content of corporate social responsibility, CSRCs explore the internal spirit and external behavior of corporate social responsibility from the perspective of organizational characteristics. Therefore, CSRCs can exert a more prominent impact on employees’ attitudes and behaviors [8]. Previous research has shown that corporate social responsibility can create a good organizational culture and moral climate, which are of great value for cultivating the moral values of employees and reducing unethical behavior [9]. EGB is a specific behavior of individual understanding, consciously accepting, and actively participating in the sustainable development of the organization [10]. However, as a strategic system for the sustainable development of the organization, little research has been carried out on the possible influence of CSRCs on EGB. In other words, can CSRCs promote EGB? Therefore, the first aim of this study was to examine the impact of CRSCs on EGB.

Affective events theory suggests that stable work environment features lead to the experience of work events and trigger individual emotional reactions, and, in turn, these environmental features affect individual attitudes and behaviors [11]. Therefore, CSRCs, as environmental features of the workplace, may influence employees’ affective responses. Moreover, CSRCs, in a moral situation, can also induce emotions. Many studies have confirmed that individual perception and participation in moral-related tasks can lead to emotional activities [12,13]. Organizational pride (OP) is defined as individuals experiencing happiness and self-esteem from organizational membership. It represents an emotional mechanism for individuals to associate organizational membership with self-concept [14]. OP is a common emotional experience of employees in the workplace and is also an important antecedent variable of employee behavior. Based on this, we applied OP to examine how CSRCs stimulate the emotional path of EGB.

In addition, Robertson and Barling [15] proposed that environmental passion is also a positive environmental-related emotion, accompanied by a strong tendency to participate in pro-environmental behavior. In other words, environmental passion is a higher spiritual pursuit, lasting pleasure, and positive tendency inspired by environmental activities. Owing to the contagious nature of emotions, members’ environmental passion in an organization easily cross-infects and forms an environmental passion climate (EPC) within the organization. Then, EPC will encourage organization members to adopt pro-environmental behavior [16]. In summary, we also explored how EPC influences the relationship between CSRCs, EGB, and OP.

In summary, based on affective events theory, we built a moderated mediation model to test how CSRCs affect EGB and explore the roles of OP and EPC in this process.

## 2. Theory and Hypothesis

### 2.1. Corporate Social Responsibility Characteristics and Employee Green Behavior

EGB is a workplace-specific form of pro-environmental behavior [17], which includes both voluntary EGB and required EGB. Voluntary EGB includes green behavior involving personal initiatives that exceed organizational expectations (e.g., prioritizing environmental interests, advocating for environmental programs and policies, participating in lobbying and activities, and encouraging others). Required EGB includes green behavior performed within the context of employees’ required job duties (e.g., complying with organizational policies, creating sustainable products and processes, and conserving resources). Therefore, EGB emphasizes that employees actively or passively fulfill their social responsibilities to the environment [18].

Prior studies have explored the impact of corporate social responsibility on pro-environmental behavior in organizational contexts from the perspectives of social exchange theory, organizational identification theory, and cue consistency theory [19,20,21]. For example, Slack et al. confirmed the relationship between corporate social responsibility and employees’ engagement with corporate social responsibility [22]. Furthermore, Ahmed et al. found that corporate social responsibility promotes EGB through a fully mediating role in well-being [23]. Moreover, De Roeck et al. found that perceived corporate social responsibility had a significant positive impact on employees’ social responsibility behavior (green behavior and social behavior) [20].

CSRCs represent the resource characteristics of the organization. They can strengthen the creation of a corporate social responsibility climate and enhance corporate social responsibility performance through the implementation of organization target responsibility standards, thus realizing the achievement of corporate social responsibility. In addition, CSRCs play an important role in the change in employees’ attitudes and behaviors [24]. Moreover, when employees are participants, executors, and observers of corporate social responsibility practices, EGB is easily affected by organizational characteristics, such as colleagues’ initiatives, organizational systems, ethical climate, and social norms [25]. Therefore, we propose that CSRCs have a direct impact on EGB.

CSRCs, despite having a positive impact on the required EGB, are also the intrinsic nature of corporate social responsibility. In other words, CSRCs go beyond the focus on the content of corporate social responsibility and are a kind of scarce, heterogeneous moral resource [7]. Therefore, organizations need to consider the cultural characteristics, values, and codes of conduct when fulfilling their social responsibilities. Properly using CSRCs enables an organization to seize the moral high ground. This change affects stakeholders, including employees’ recognition of value, and employees will then take the initiative to act in favor of the organization’s development. Therefore, CSRCs positively affect voluntary EGB. Based on the discussion above, we propose the following.

**Hypothesis** **1:** *CSRCs have a positive impact on required EGB.*

**Hypothesis** **2:** *CSRCs have a positive impact on voluntary EGB.*

### 2.2. Organizational Pride and Employee Green Behavior

Moral emotion is a kind of emotion related to personal or social interests and well-being when individuals evaluate their own or others’ behaviors according to social norms or codes of conduct [26,27,28]. When behavior conforms to social norms and benefits others, individuals will feel positive moral emotions, such as pride [29]. Moral emotion is the emotional experience of altruism and pro-sociality [30]. Moreover, the pro-sociality of moral emotion goes beyond the dimension of emotional valence. Both the positive and negative moral emotions generated by the evaluation of their own or others’ behavior have important pro-sociality and altruism [31]. Affective event theory can effectively explain the “black box” of the relationship between work situations and organizational members’ behavior.

Bachrach and Jex [32] pointed out that employees with higher levels of positive emotions extend their job responsibilities and scope. These changes encourage employees to show more EGB, which can benefit the organization in daily work. The positive moral emotion of pride can strengthen and motivate the continuation of and increase in personal social value behaviors. For example, participants’ pride in previous pro-environmental behavior predicted continued engagement in environmental donations and voluntary behavior [33].

CSRCs indicate that the organization cares for internal and external stakeholders. It not only sends a signal to employees that the organization is working toward a higher goal but is also an important way to establish a positive social image of the organization. When the outside believes that the organization has a good reputation and a sense of social responsibility, employees will feel a high-quality external relationship between the organization and society. Furthermore, employees will also indirectly generate moral and valuable judgments about the organization. Therefore, employees will be proud of their membership in the organization and then produce more EGB [34]. EGB is a specific behavior of prosocial behavior. We suggest that when CSRCs are strong, positive moral emotions of employees’ OP will be stimulated. Based on the discussion above, we proposed the following.

**Hypothesis** **3:** *Moral emotions (OP) mediate the association between CSRCs and required EGB.*

**Hypothesis** **4:** *Moral emotions (OP) mediate the association between CSRCs and voluntary EGB.*

### 2.3. Moderating Effect of Environmental Passion Climate

Robertson and Barling [15] proposed that environmental passion is a positive environment-related emotion. It is often accompanied by a strong tendency to participate in environmental protection behaviors. In other words, environmental passion is a higher spiritual pursuit, lasting pleasure, and positive tendency inspired by environmental protection activities. Similarly, it is also a positive moral emotion. Owing to the contagious nature of emotions, the environmental passion of employees within an organization is easily cross-infected and forms an EPC within the organization. Then, EPC will promote organization members to enact pro-environmental behavior [16]. EPC, as a source of information about the environment, plays an important role as reference information when organizational members make cognitive judgments about events [35]. In other words, EPC can help employees better explain pro-environmental behavior. Therefore, the following hypothesis is suggested.

**Hypothesis** **5:** *EPC plays a moderating role in the impact of OP on required EGB.*

**Hypothesis** **6:** *EPC plays a moderating role in the impact of OP on voluntary EGB.*

**Figure 1:**
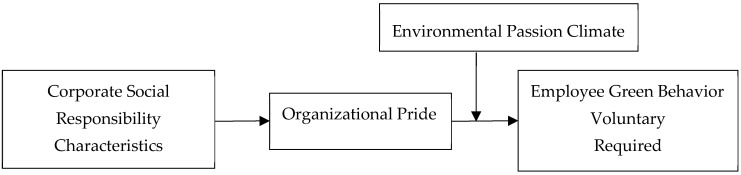
Theoretical model.

In summary, this article proposes a moderated mediation model, as shown in Figure 1. Thus, we proposed the following hypothesis:

**Hypothesis** **7:** *EPC moderates the indirect impact of CSCs on required EGB via EPC.*

**Hypothesis** **8:** *EPC moderates the indirect impact of CSCs on voluntary EGB via EPC.*

## 3. Methods

### 3.1. Data Collection

The current study adopted a cross-sectional research design to collect self-reported data from employees in China. As suggested by Gorsuch [36], the ratio of the sample size to the number of items should be 5:1. Accordingly, this study included 30 items, necessitating a minimum of 150 samples (30 × 5). Given that structural equation modeling (SEM) requires a larger sample size [37], we ultimately issued twice the recommended amount, i.e., 300 questionnaires.

From June to July 2023, the web link to the online questionnaire, which was designed using the Sojump platform (http://www.wjx.cn), was disseminated to the participants through the Sojump platform and WeChat app. Sojump is a professional platform for specialized data collection in China. It was randomly sent to participants across the country through the platform to further reduce the impact of regional differences. Moreover, we limited the industries of the participants through the platform, mainly focusing on oil, coal, and other industries that pay more attention to green issues. To improve the quality of the questionnaire, we set up a reward of RMB 5 for every participant. The first page of the survey outlined this study’s aims, potential risks, and benefits. It was made clear to the participants that the survey was conducted solely for academic purposes. The participation was voluntary, and no personal data were collected. Finally, 267 usable responses were received, corresponding to a response rate of 89%.

There were 139 males and 128 females who participated effectively in the survey, and there were more people between the ages of 21 and 30. Most of the employees had a bachelor’s degree (74.9%), and 51.3% had 5–10 years of work experience.

Written informed consent was first obtained from the subjects after explaining the purpose and significance of this study at the beginning of the questionnaires. Subjects were guaranteed that participation was voluntary and anonymous. After that, the survey instrument, including demographic questions, was distributed to each subject. If they felt uncomfortable with the questions, they could stop filling in the questionnaire at any time. When they finished the questionnaire, participants were offered a subject fee through WeChat to compensate them for their time and assistance. All the data were analyzed anonymously.

### 3.2. Measures

All measurements used a mature Chinese version of the questionnaire, with good reliability and validity. However, the scales of EGB and OP were cited in English articles and subsequently translated into Chinese. To avoid distortion in the translation, two professionally independently translated the scales back to English and compared them with the original English version of the paper. As presented in “Appendix A”, all items were administered on a 6-point Likert scale (1 = strongly disagree; 6 = strongly agree).

EGB was measured using six items from Bissing-Olson’s study [38], which consisted of two dimensions: required EGB and voluntary EGB. For example, “I fulfilled responsibilities specified in my job description in environmental-friendly ways.” Cronbach’s α for EGB was 0.784. The scale has been used widely in China and demonstrated to be reliable and valid [39].

CSRCs were measured using 12 items from Xi’s study [24]. The scale included three dimensions: corporate social responsibility culture, corporate social responsibility climate, and corporate social responsibility performance. For example, “Our organization attached importance to the interests and development of employees.” Cronbach’s α for CSRC was 0.844.

OP was measured using the six-item scale, developed by Gouthier and Rhein [40], which is divided into two dimensions: emotional pride and attitudinal pride. For example, “I have a feeling of joy to be a part of this company.” Cronbach’s α for OP was 0.796. The scale has been used widely in China and demonstrated to be reliable and valid [41].

EPC was measured using the six-item scale from Wang and Peng’s research [16], which was adapted from the Harmony Environmental Passion Scale developed by Robertson and Barling. The adapted scale could better reflect the environmental passion at the group level in the workplace. For example, “We are passionate about the environment.” Cronbach’s α for EPC was 0.805.

The demographic variables were gender, age, education level, and working years. Previous research has proved that these factors affect the green behavior of employees [42].

### 3.3. Data Analysis

SPSS 25.0 and Mplus 8.0 were utilized for data analysis. First, we used Mplus 8.0 to perform confirmatory factor analysis on the four variables involved in this study. Second, we presented the means, standard deviations, and correlation values between the variables with SPSS25.0. Finally, the SEM was used to test the hypothesis.

## 4. Results

### 4.1. Common Method Bias Test

We controlled for the common method bias issue in different ways. First, an anonymous survey and disrupted item order were used to keep the participants from thinking in the process of filling out the questionnaire. Subsequently, the common method variance was tested by a Harman single-factor test. The results show that the cumulative variation explained by the first factor was only 29.47% (less than 40%), revealing that there was no serious common method variance in this study.

### 4.2. Confirmatory Factor Analysis

We examined the measurement validity of our four constructs using confirmatory factor analysis (CFA) with Mplus 8.0. As Table 1 depicts, our theoretical four-factor model had an excellent fit to the data and was superior to simpler representations of the data (χ^2^ = 632.014, DF = 371, RMSEA = 0.050, CFI = 0.910, TLI = 0.901, and SRMR = 0.055).

### 4.3. Descriptive Statistical Analysis

Table 2 details the means, standard deviations, and correlations. The results show that CSRCs were significantly positively correlated with OP, EPC, required EGB, and voluntary EGB; that OP was significantly positively correlated with EPC, required EGB, and voluntary EGB; that EPC was significantly positively correlated with required EGB and voluntary EGB; and that required EGB was significantly positively correlated with voluntary EGB.

### 4.4. Hypothesis Testing

First, after controlling the control variables, the direct effect of CSRCs on required EGB (b = 0.314, SE = 0.088, and *p* < 0.01) and the direct effect of CSRCs on voluntary EGB (b = 0.309, SE = 0.101, and *p* < 0.01) was significant. Thus, Hypotheses 1 and 2 were supported.

**Figure 2:**
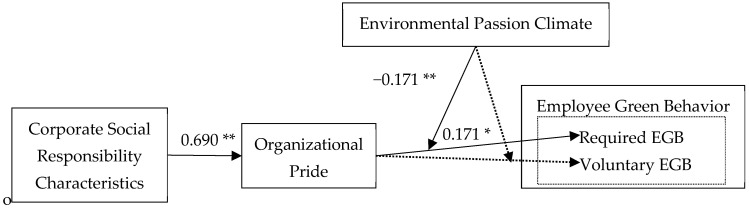
Results of multilevel path analysis. * *p* < 0.05, ** *p* < 0.01. The solid lines were supported, and the dashed lines were not supported.

Second, we constructed an SEM for item-by-item testing. As shown in Figure 2, CSRCs had a significant positive correlation with OP (b = 0.690, SE = 0.054, and *p* < 0.01), and OP had a significant positive correlation with required EGB (b = 0.171, SE = 0.085, and *p* < 0.05). However, OP had no significant correlation with voluntary EGB (b = 0.031, SE = 0.087, and *p* = 0.717). We used bootstrap to further test the mediating effect of OP, and repeated sampling was set to 2000. The results show that all the bootstrap bias-corrected 95% CIs excluded zero, revealing that the indirect effects of CSCR on required EGB through OP were significant (b = 0.166, SE = 0.053, *p* < 0.05, and 95% CI = [0.102, 0.235]). Thus, Hypothesis 3 was supported, but Hypothesis 4 was not.

Subsequently, we tested the moderating effect of EPC. The results in Figure 2 show that the interaction between OP and EPC had a significant negative impact on required EGB (b = −0.171, SE = 0.075, and *p* < 0.05). In other words, there existed a certain substitution effect between EPC and OP. However, the interaction between OP and EPC had no significant impact on voluntary EGB (b = −0.147, SE = 0.093, and *p* = 0.113). Therefore, Hypothesis 5 was supported, while Hypothesis 6 was not.

**Figure 3:**
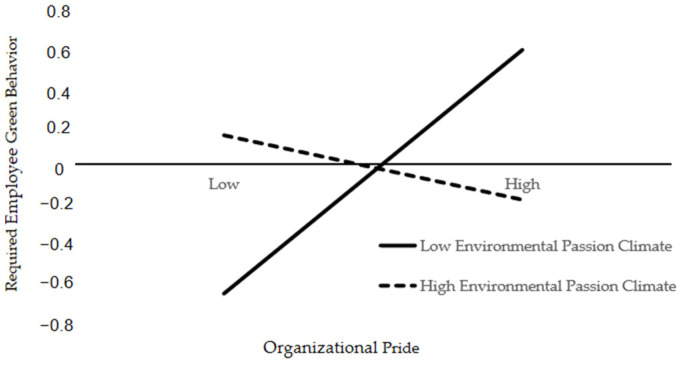
The moderating effect of EPC on OP and required EGB.

To further explain the essence of the interaction between OP and EPC more clearly, we divided OP into high and low groups according to the average addition and subtraction of a standard deviation (Figure 3). A simple slope test showed that when EPC was high, the effect of OP on required EGB was not significant (b = −0.096, SE = 0.138, and *p* = 0.485). However, when the EPC was low, the effect of OP on the required EGB was significant (b = 0.43).

Finally, we tested the moderated mediation. As shown in Table 3, the indirect effect of CSRCs on required EGB through OP significantly varied across levels of EPC, and the bootstrap bias-corrected 95% CIs excluded zero (b = −0.066, SE = 0.095, and 95% CI = [−0.636, −0.023]). Specifically, the indirect effect of CSRCs on required EGB through OP was not significant when EPC was high (b = −0.066, SE = 0.095, and 95% CI = [−0.265, 0.083]). However, the indirect effect of CSRCs on required EGB through OP was significant when EPC was low (b = −0.066, SE = 0.095, and 95% CI = [−0.636, −0.023]). Hypothesis 7 was supported.

## 5. Discussion

### 5.1. Interpreting the Findings

Based on the affective events theory, we explored the impact of CSRCs on EGB. Furthermore, we focused on the mediating role of OP and the moderating role of EPC. In other words, we constructed an SEM of the impact of CSRCs on EGB, drawing the following conclusions.

CSRCs positively affect required EGB. EGB is closely related to the organizational environment. In other words, whether it is organizational rules and regulations, cultural climate, or organizational performance, they all influence employees’ mentality and behavior [6]. Through empirical research, we confirmed that an organization should assume social responsibilities at a higher level and actively create a good organizational culture climate, which can promote the generation of required EGB. Moreover, OP plays a mediating role in the relationship between CSRCs and the required EGB. This indicates that an organization’s participation in corporate social responsibility activities, such as showing environmental behavior and caring for the environment, is an important source for employees’ OP. OP is a valuable psychological resource that can inspire employees to implement environmental actions, provide environmental help, and produce more EGB.

The impact of CSRCs on voluntary EGB is not significant, probably because voluntary EGB is more related to employees’ self-green awareness. The organizational performance and organizational culture climate can promote employees to complete the required EGB more actively. However, as a moral emotion, OP affects employees’ current behavior, rather than their awareness.

EPC is the boundary condition of the impact of CSRCs on required EGB. We found that the interaction between OP and EPC had a significant negative impact on required EGB (b = −0.171, SE = 0.075, and *p* < 0.05). In other words, there existed a certain substitution effect between EPC and OP. However, the interaction between OP and EPC had no significant impact on voluntary EGB. Moreover, the indirect effect of CSRCs on required EGB through OP was only significant when EPC was low. This indicates that the impact of CSRCs on OP and required EGB needs to be guaranteed by specific conditions.

### 5.2. Theoretical Implications

First, based on the perspectives of internal employees, this study examined the relationship between CSRCs and EGB, which is helpful for further detailed research and processing research on CSR. Moreover, we broke through the lack of the micro-individual level in previous CSR research by conducting theoretical research on CSR from the perspective of employees’ moral emotions, especially the limitations of the impact on employees’ psychology and behavior. In addition, the empirical research method was used to examine the proposal that CSRCs at the organizational level affect EGB at the individual level. The study results not only promote the combination of corporate social responsibility and organizational behavior theory but also reinforce and deepen the traditional oriental management wisdom of “people-oriented” and “morality first”.

Second, we found that CSRCs positively influenced EGB. This finding finds new influencing factors for the study of EGB, enriches the research on EGB, and also makes corporate social responsibility better implemented.

Finally, this study regarded EGB as a specific ethical behavior in the field of moral decision-making research. This makes up for the lack of empirical research in the existing research owing to the difficulty of measuring moral behavior, and it enriches the research results of moral decision making. Previous research on moral decision making in organizations has been based on a rational perspective, ignoring the stimulating effect of emotional factors. Thus, this study examined the psychological mechanism of the effect of CSRCs on EGB from the perspective of moral emotions and expanded the research content of moral decision-making theory.

### 5.3. Management Implications

Our findings provide some management implications for organizations to take measures to promote EGB. Organizations should pay more attention to public participation and actively promote and implement relevant environmental regulations. These measures will enable employees to better understand and comply with social norms and to better promote social progress. By creating a healthy, caring, and motivating environment, organizations can increase employees’ organizational sense of belonging, stimulate employees’ respect for the organization, and cultivate employees’ team cohesion, thus promoting the development of their organizations. In addition, organizations should encourage employees to participate in the “Planting Hope Festival, environmental innovation essay contests, and speech contests. These activities can make employees more aware of the corporate social responsibility culture, enhance employees’ self-green awareness, and increase contributions to the environmental performance of organizations. To promote organizational development better, organizations need to actively carry out publicity and communication activities, as well as integrate the organizations’ core values into the organizations’ overall climate. In addition, the government should actively encourage organizations to assume more social responsibilities to better enable organizations to become beneficiaries of the public interest.

EGB can greatly contribute to organizations’ environmental and economic growth and is an important part of organizations’ long-term stable growth. Our findings suggest that organizations should take steps to promote EGB and should strengthen their daily management. First, organizations need to establish an effective environmental protection strategy and a set of strict industry guidelines so that every employee can make effective environmental contributions according to their own position. Second, it is necessary for organizations to emphasize the consideration of employees’ interests in daily operations so that every employee can truly understand the environmental protection policy of the organization. Third, organizations must vigorously promote environmental education and provide each employee with sufficient learning resources. This can help employees master relevant environmental knowledge and effectively handle various environmental challenges. In addition, it is necessary to organize a variety of useful community activities to stimulate employees’ sense of environmental responsibility and to make them keen to adopt a sustainable development approach.

### 5.4. Limitations and Future Directions

There were some limitations in our sample selection. First, the age, working years, and industry of the participants were relatively concentrated. Especially in the industry, different industries have different demands for employees. Although we limited the industry, there were still many typical industries related to the environment that were not involved, such as JD Logistics, Meituan Takeaway, and other relevant departments. This study of energy, recycling, and other energy degrees of organization-related information was limited, potentially leading to deviations in the collected data. The reason why the mediating role of negative moral emotions in this study was not confirmed is also because the number of sample companies selected was insufficient. In view of the above situation, more sample data from typical green industries can be added, and the sample size can be expanded to further test the hypothesis model to determine the causality of this study in the future. This approach can improve the scientific and universal nature of this research. It can also help further examine the role of negative moral emotions.

Second, the data sources of this study were taken from the same time point. In other words, the data were cross-sectional rather than longitudinal. However, this design does not permit the clarification of causal relationships. Due to the lack of inter-temporal research and the short research time, voluntary EGB with autonomy could not be well measured. This may be the reason why there was no significant correlation between OP and voluntary EGB. Furthermore, the defects of cross-sectional research also made it impossible for us to effectively test the transformation process from required EGB to voluntary EGB. Therefore, future research should use longitudinal data to track how CSRCs affect EGB, and how required EGB driven by external motivation translates into voluntary EGB by employees’ internal motivation. This will better reflect the causal relationship between variables and encourage organizations to implement green behaviors.

## Figures and Tables

**Table 1:** Results of confirmatory factor analysis.

Model	χ^2^	df	△χ^2^ (Δdf)	RMSEA	SRMR	CFI	TLI
CSRCs; OP; EPC; EGB	632.014	371	—	0.050	0.052	0.911	0.901
CSRCs + OP; EPC; EGB	778.493	374	146.479(3) ***	0.064	0.061	0.853	0.840
CSRCs + EPC; OP; EGB	673.608	374	41.594(3) ***	0.055	0.055	0.891	0.882
CSRCs + EGB; OP; EPC	722.804	374	90.790(3) ***	0.059	0.059	0.873	0.862
CSRCs; OP + EPC, EGB	772.137	374	140.123(3) ***	0.060	0.063	0.855	0.843
CSRCs; OP + EGB; EPC	724.693	374	92.679(3) ***	0.059	0.059	0.872	0.861
CSRCs; OP; EPC + EGB	656.018	374	24.004(3) ***	0.055	0.053	0.897	0.889
CSRCs + OP + EPC + EGB	862.219	377	230.205(3) ***	0.069	0.063	0.823	0.810

*** *p* < 0.001, CSRCs = corporate social responsibility characteristics; OP = organizational pride; EPC = environmental passion climate; EGB = employee green behavior.

**Table 2:** Means, standard deviations, and correlations of variables.

Variable	1	2	3	4	5	6	7	8	9
1 Gender									
2 Age	0.002								
3 Education level	−0.035	−0.022							
4 Working years	0.099	0.698 **	−0.092						
5 CSRCs	0.054	−0.192 **	0.107	−0.117					
6 OP	−0.005	−0.094	0.109	−0.110	0.665 **				
7 EPC	0.113	−0.015	0.111	0.049	0.522 **	0.524 **			
8 Required EGB	0.014	−0.090	0.085	−0.086	0.536 **	0.507 **	0.525 **		
9 Voluntary EGB	0.073	−0.093	0.103	−0.083	0.569 **	0.516 **	0.627 **	0.531 **	
Mean	1.520	2.600	3.831	3.901	4.533	4.902	4.913	4.743	4.769
SD	0.501	0.632	0.619	1.043	0.604	0.596	0.605	0.659	0.711

N = 267, ** *p* < 0.01. CSRCs = corporate social responsibility characteristics; OP = organizational pride; EPC = environmental passion climate; EGB = employee green behavior.

**Table 3:** Analysis of moderated intermediate effects.

EPC	CSRCs → OP → EGB		
	Indirect Effect	Standard Deviation	95% CI
Low EPC	0.303	0.109	(0.076, 0.505)
High EPC	−0.066	0.095	(−0.265, 0.083)
Differences in indirect effects between high and low conditions	−0.066	0.095	(−0.636, −0.023)

CSRCs = corporate social responsibility characteristics; OP = organizational pride; EPC = environmental passion climate; EGB = employee green behavior.

## Data Availability

The datasets generated and analyzed during the current study are not publicly available but are available from the corresponding author upon reasonable request.

## Appendix A

**Corporate social responsibility characteristics**

1. The mission of our organization included the content of common development and return to the society.
2. Vision of our organization is worth fighting for.
3. Leaders in our organization set an example to promote the construction of social responsibility.
4. Our organization attached importance to the interests and development of employees.
5. Our organization encouraged employees to carry out social responsibility activities and use enterprise resources to support them.
6. Our organization organized publicity activities to promote the exchange of understanding of social responsibility.
7. The average salary level in our organization was competitive locally.
8. The education and training of employees in our organization was better than that of peers.
9. Our organization supported local cultural, educational and sports undertakings.
10. The employment opportunities of our organization gave priority to the local community.
11. Our organization donated more than their peers in the event of a major social disaster.
12. Our organization attached great importance to environmental protection.

**Organizational pride:**

1. I have a feeling of joy to be a part of this company.
2. I am proud of what the company has achieved.
3. I have the feeling that the company is doing something meaningful.
4. I feel proud to contribute to my company’s success.
5. I feel proud to work for my company.
6. I feel proud to tell others for which company I am working.

**Environmental passion climate:**

In our organization,

1. We are passionate about the environment.
2. We enjoy practicing environmentally friendly behaviors.
3. We enjoy engaging in environmentally friendly behaviors.
4. We take pride in helping the environment.
5. We enthusiastically discuss environmental issues with others.
6. We feel strongly about our environmental values.

**Employee green behavior:**

1. I adequately completed assigned duties in environmentally-friendly ways.
2. I fulfilled responsibilities specified in my job description in environmentally-friendly ways.
3. I performed tasks that are expected of me in environmentally-friendly ways.
4. I took a chance to get actively involved in environmental protection at work.
5. I took initiative to act in environmentally-friendly ways at work.
6. I did more for the environment at work than I was expected to.
